# Lumbar Puncture in Saudi Arabia: Evaluating Knowledge, Attitude and Sociodemographic Influences

**DOI:** 10.3390/healthcare14162518

**Published:** 2026-08-12

**Authors:** Eyad M. Albarrati, Mohammad A. Jareebi, Khalid I. Hakami, Jawaher S. Farji, Reema Nharri, Emtenan A. Mawkili, Ahmed Y. Najmi, Saja A. Almraysi, Mohammed A. Baowideen, Asma A. Jabrah, Yahya H. Khormi, Farjah H. Algahtani, Majed A. Ryani, Ahmed A. Bahri, Ghazi I. Al Jowf

**Affiliations:** 1 Faculty of Medicine, Jazan University, Jazan 45142, Saudi Arabia; hakamikhalid23@gmail.com (K.I.H.); emtnan3.13@gmail.com (E.A.M.); ahmednajmiofficial@gmail.com (A.Y.N.); sajaalasiri1@gmail.com (S.A.A.);; 2 Department of Family and Community Medicine, College of Medicine, Jazan University, Jazan 45142, Saudi Arabia; majedryani@gmail.com (M.A.R.); dr.bahri2010@gmail.com (A.A.B.); 3 Faculty of Medicine, King Abdulaziz University, Jeddah 21589, Saudi Arabia; mbaowideen@gmail.com; 4 Department of Surgery, Faculty of Medicine, Jazan University, Jazan 45142, Saudi Arabia; khormins@gmail.com; 5 Oncology Center, Chair of Epidemiology and Public Health Research, Faculty of Medicine, King Saud University/King Saud Medical City, Riyadh 12373, Saudi Arabia; falgahtani@ksu.edu.sa; 6 Department of Public Health, College of Applied Medical Sciences, University Medical Clinics Complex, King Faisal University, Al Hofuf 37912, Saudi Arabia; galjowf@kfu.edu.sa

**Keywords:** lumbar puncture, health literacy, misconceptions, Saudi Arabia, sociodemographic determinants, cross-sectional study

## Abstract

**Background/Objectives**: Lumbar puncture (LP) is an essential diagnostic and therapeutic procedure in neurological practice. Despite its clinical value, misconceptions and fear may influence its acceptance among the public. This study aimed to assess knowledge, attitudes, and misconceptions regarding LP among adults in Saudi Arabia and to examine associated sociodemographic factors. **Methods**: A cross-sectional study was conducted between June 2025 and January 2026 using a structured, self-administered online questionnaire adapted from a previously validated instrument. Adults aged ≥18 years residing in Saudi Arabia were recruited via convenience sampling through open social media channels, so the sample is self-selected rather than population-representative. Knowledge and attitude scores were categorized into defined levels, and multivariable regression analyses were performed to identify independent predictors. **Results**: A total of 1267 participants were included. Knowledge of LP was limited (mean 6.1 ± 3.9 of 18), with 905 (71%) scoring poor and only 362 (29%) demonstrating good knowledge; misconceptions about complications such as urinary incontinence and erectile dysfunction were common. Attitudes appeared favorable, with 1182 (93%) reaching the pre-specified 50% threshold, but this cut-off is lenient because an entirely neutral respondent scores 60% on a summed five-point scale, and only 692 (55%) agreed they would sign consent if their physician recommended LP. Knowledge and attitude were weakly but significantly related (r = 0.21, 95% CI 0.16 to 0.26; adjusted β = 0.25 per additional correct item, *p* < 0.001). Higher education, student status, healthcare occupation, and higher income predicted better knowledge, and male sex predicted lower knowledge (all *p* < 0.05), whereas sociodemographic factors were weakly associated with attitude. **Conclusions**: Among self-selected online respondents in Saudi Arabia, knowledge of lumbar puncture was limited while attitudes were comparatively favorable, and the two were weakly but positively related. Targeted educational interventions are warranted to address misconceptions, enhance public awareness, and support informed decision-making within the healthcare system.

## 1. Introduction

Lumbar puncture (LP), commonly known as a spinal tap, is a medical procedure performed to obtain cerebrospinal fluid (CSF) for diagnostic and therapeutic purposes in various neurological conditions. It remains an essential procedure for confirming or excluding serious disorders affecting the central nervous system, such as meningitis, encephalitis, and subarachnoid haemorrhage [1,2]. Beyond diagnosis, LP also serves therapeutic roles, including the administration of intrathecal chemotherapy and spinal anaesthesia, and the measurement or relief of intracranial pressure in select neurological conditions. Early and accurate diagnosis through LP can significantly improve patient outcomes and reduce morbidity and mortality, particularly in time-sensitive conditions such as bacterial meningitis, where delayed diagnosis is strongly associated with worse neurological outcomes [1].

Although LP is generally considered safe when performed by trained personnel, it remains an invasive procedure and may be associated with minor complications such as post-procedural headache, back pain, and localized bleeding. Rare but serious complications, including infection, nerve injury, and brain herniation due to changes in intracranial pressure, have also been reported [2]. Despite its established clinical importance and long history of use, fear of complications and insufficient understanding of its necessity remain major factors contributing to patient reluctance and refusal [3]. Misconceptions and anxiety continue to significantly influence acceptance of LP in clinical practice, and patients often overestimate the likelihood of rare complications while underestimating or misunderstanding the more common, self-limited ones [3].

International studies have demonstrated considerable variability in knowledge and attitudes toward lumbar puncture across different healthcare systems and cultural contexts. Across different populations, concerns regarding pain and safety remain among the most common reasons cited for refusal [4]. Recent evidence also highlights that limited health literacy and cultural beliefs continue to influence acceptance of LP in low- and middle-income countries, where misinformation often persists despite medical reassurance and thorough counselling [5,6]. These findings suggest that knowledge deficits are not confined to any single healthcare system but represent a broader, global challenge in public understanding of invasive diagnostic procedures.

In Saudi Arabia, studies indicate insufficient knowledge and variable attitudes toward lumbar puncture among the general population [7,8,9]. For instance, prior regional research showed that a majority of participants had limited knowledge regarding the procedure, with sociodemographic factors such as age, education level, and healthcare exposure significantly influencing awareness and perceptions of LP [7,10]. These regional findings mirror international patterns, suggesting that despite differences in healthcare infrastructure, the underlying drivers of poor LP literacy, namely limited exposure to the procedure and reliance on informal or unreliable sources of information, may be broadly similar across populations.

Despite the procedure’s clinical value, previous Saudi studies have largely been confined to single regions, with most focusing on caregivers of pediatric patients, and have examined either knowledge or attitudes rather than both together with their sociodemographic correlates. The present study is therefore framed as a large-scale replication and extension of Aldayel et al. [8], applying the same instrument in a sample roughly twice the size and drawn from all administrative regions rather than Riyadh alone, and adding a direct within-individual analysis of the knowledge-attitude relationship that earlier Saudi work reported only as a comparison of aggregate percentages.

This study therefore aimed to evaluate knowledge, attitudes, and misconceptions regarding lumbar puncture among adults in Saudi Arabia and their association with sociodemographic factors, in order to inform targeted educational programs and support informed decision-making in the Saudi healthcare context.

## 2. Materials and Methods

### 2.1. Study Design and Setting

This study employed a cross-sectional design to assess knowledge, attitudes, and misconceptions regarding lumbar puncture among adults residing in Saudi Arabia. The target population comprised all individuals aged 18 years and above, encompassing a broad range of sociodemographic backgrounds to provide meaningful insights into public perceptions of the procedure. Individuals who were unwilling or unable to provide informed consent were excluded from participation.

### 2.2. Sampling and Sample Size Calculation

Participants were recruited using convenience sampling through online platforms, selected for its practicality, cost effectiveness, and ability to reach a geographically dispersed nationwide sample within the available timeframe and resources. This approach does not yield a probability-based sample, although it has been widely employed in similar knowledge and attitude studies across the region. Recruitment through social media inherently favors individuals who are younger, educated, and digitally active, and because the questionnaire link was shared openly rather than sent to an enumerated list, there is no sampling frame and no invitation denominator, so a response rate cannot be computed, and coverage bias cannot be quantified.

Based on the total population aged 18 years and above in Saudi Arabia (22,091,634) according to the latest census [11], and using a 95% confidence level and a 5% margin of error, the minimum required sample size was calculated as 385 participants, using the standard formula for proportions:

$$n^{0}=\frac{Z^{2}\cdot p\cdot (1-p)}{e^{2}}$$

where Z denotes the standard score corresponding to the selected confidence level (1.96 for a 95% CI), p indicates the anticipated prevalence or proportion (0.5 for a conservative estimate), and e represents the allowable margin of error. Allowing 15% incomplete or unusable responses, the target increased to 453. Ultimately, 1267 responses were collected, exceeding this threshold and ensuring adequate power for multivariable regression with numerous covariates. This calculation should be read as a minimum precision target rather than a guarantee of national representativeness, since the confidence level and margin of error assume probability sampling, which was not used here.

### 2.3. Data Collection Tool

Data were collected through a self-administered questionnaire in Arabic, adapted from a previously validated tool by Wan Sulaiman et al. (2018) [1], which reported acceptable internal consistency (Cronbach’s α = 0.701). The Arabic version was used with the permission of Aldayel et al. (2019) [8] and reviewed by a senior neurologist and the institutional translation committee.

The questionnaire comprised four sections. The first gathered demographic information, and the second assessed previous experience, awareness of lumbar puncture, and fear of needles. The third evaluated knowledge through 18 questions on the nature, indications, and complications of the procedure. The fourth assessed attitudes using 10 questions, each answered on a five-point Likert scale scored from 1 (strongly disagree) to 5 (strongly agree), with the seven negatively worded items reverse-scored so that higher values consistently denote a more favorable attitude; item scores were summed to give a total ranging from 10 to 50.

Raw scores for both scales were converted to percentages of the maximum and classified as poor (<50%), moderate (50–75%), or excellent (>75%), following the scheme used by the instrument’s developers [1]. The item on lying flat after the procedure was keyed with “no” as correct, consistent with evidence that routine bed rest does not prevent post-dural puncture headache [12]. These cut-offs are inherited from the source instrument rather than derived from any external criterion, and on a summed five-point scale they are lenient, because the arithmetic floor is 20% and a respondent answering neutrally to every item scores 60%. Attitude categories should therefore be read as relative positions within this sample rather than validated thresholds, alongside the item-level distribution In the present sample, internal consistency was 0.83 (KR-20) for the knowledge items and 0.66 (Cronbach’s alpha) for the attitude items.

### 2.4. Data Collection Process

Data collection occurred between June 2025 and January 2026 using a self-administered online questionnaire. The questionnaire was piloted with 20 participants to assess its clarity before it was finalized. The questionnaire link was distributed through various social media platforms such as WhatsApp, Telegram, X (formerly Twitter), and Snapchat. An explanatory page detailed the study objectives, confidentiality protections, and participants’ rights. Responses were monitored regularly for completeness, and incomplete or incoherent entries were excluded from the final dataset.

### 2.5. Statistical Analysis

Following data collection, the raw dataset was transferred to Microsoft Excel (Microsoft Corporation, Redmond, WA, USA) for preliminary cleaning, error detection, and validation. Three image-based items were unanswered by one participant and scored as incorrect; no data were imputed. Statistical analyses were performed in R (version 4.2.3; R Foundation for Statistical Computing, Vienna, Austria). Descriptive statistics including frequencies, means, percentages, and standard deviations summarized participant characteristics and outcome variables.

Multiple linear regression models were fitted to identify factors associated with knowledge and with attitude, using covariates specified a priori. Because the occupation reference category (unemployed/student) duplicated two employment categories, employment and occupation were combined into a single role variable with unemployed respondents as the reference. Model assumptions were assessed using residual plots, and variance inflation factors were examined for multicollinearity. The relationship between knowledge and attitude was examined by Pearson correlation, by cross-tabulation with a chi-square test, and by entering the knowledge score as an explanatory variable in the attitude model. Overall model fit was summarized by the coefficient of determination (R^2^), the overall F test, and the Akaike information criterion (AIC), and the contribution of the knowledge score to the attitude model was assessed by a partial F test comparing nested models. All tests were two-tailed with a significance threshold of α = 0.05, and results were reported as beta coefficients (β) with 95% confidence intervals (CIs).

### 2.6. Ethical Considerations

All necessary official permissions were obtained prior to data collection, including ethical review and approval granted by the Jazan University Research Ethics Committee (REC) (Reference No.: REC-46/12/1551, Dated 30 May 2025). Participation was completely voluntary, and electronic informed consent was mandated. The study adhered to the Strengthening the Reporting of Observational Studies in Epidemiology (STROBE) guidelines [13], see Supplementary Materials.

### 2.7. Use of Generative Artificial Intelligence

A generative AI tool (ChatGPT-5 (OpenAI)) was used solely for language editing. The study design, literature synthesis, data extraction, analysis, and interpretation were entirely conducted by the authors.

## 3. Results

### 3.1. Sociodemographic and Clinical Characteristics of Participants

The study enrolled 1267 participants, the majority of whom were females (n = 722, 57%) while 545 (43%) were males. Most of the participants were Saudi nationals (n = 1156, 91%), while 111 (9%) were non-Saudi. The mean age of the participants was 30 ± 12 years, with a range of 18 to 73 years. The majority were single (n = 784, 62%) and most resided in urban areas (n = 960, 76%). Regarding education, most participants had a bachelor’s degree or diploma (834, 66%), while 28% (n = 349) had a high school education or lower, and 84 (7%) had postgraduate qualifications. In terms of employment, half of the participants were students (n = 631, 50%), while 404 (32%) were employed, and 405 (32%) reported a monthly household income of <5000 SAR (Table 1).

### 3.2. Clinical Profile of Participants

Table 2 shows that a minority of participants reported chronic medical conditions. Diabetes mellitus was present in 106 (8%) participants, and hypertension was also reported in 100 (8%). Hyperlipidemia was observed in 130 (10%), while asthma was reported in 94 (7%) participants. Less common conditions included hypothyroidism (58, 5%) and hernia (39, 3%). Sickle cell anemia and hyperthyroidism were each reported in 27 (2%) participants, while rheumatoid arthritis was present in 25 (2%). Thalassemia was the least reported condition, affecting only 12 (1%) participants.

### 3.3. LP Knowledge and Attitude Characteristics

Regarding subjective knowledge and experience with LP, 610 (48%) participants had heard of lumbar puncture. Personal experience with LP was limited, reported by only 33 (3%) participants, while 170 (13%) reported that a family member or friend had experience with the procedure. Fear of needle injection in the hand was reported by 375 (30%) participants, while fear of needle injection in the back was considerably higher, reported by 877 (69%) participants (Appendix A Table A1).

**Figure 1:**
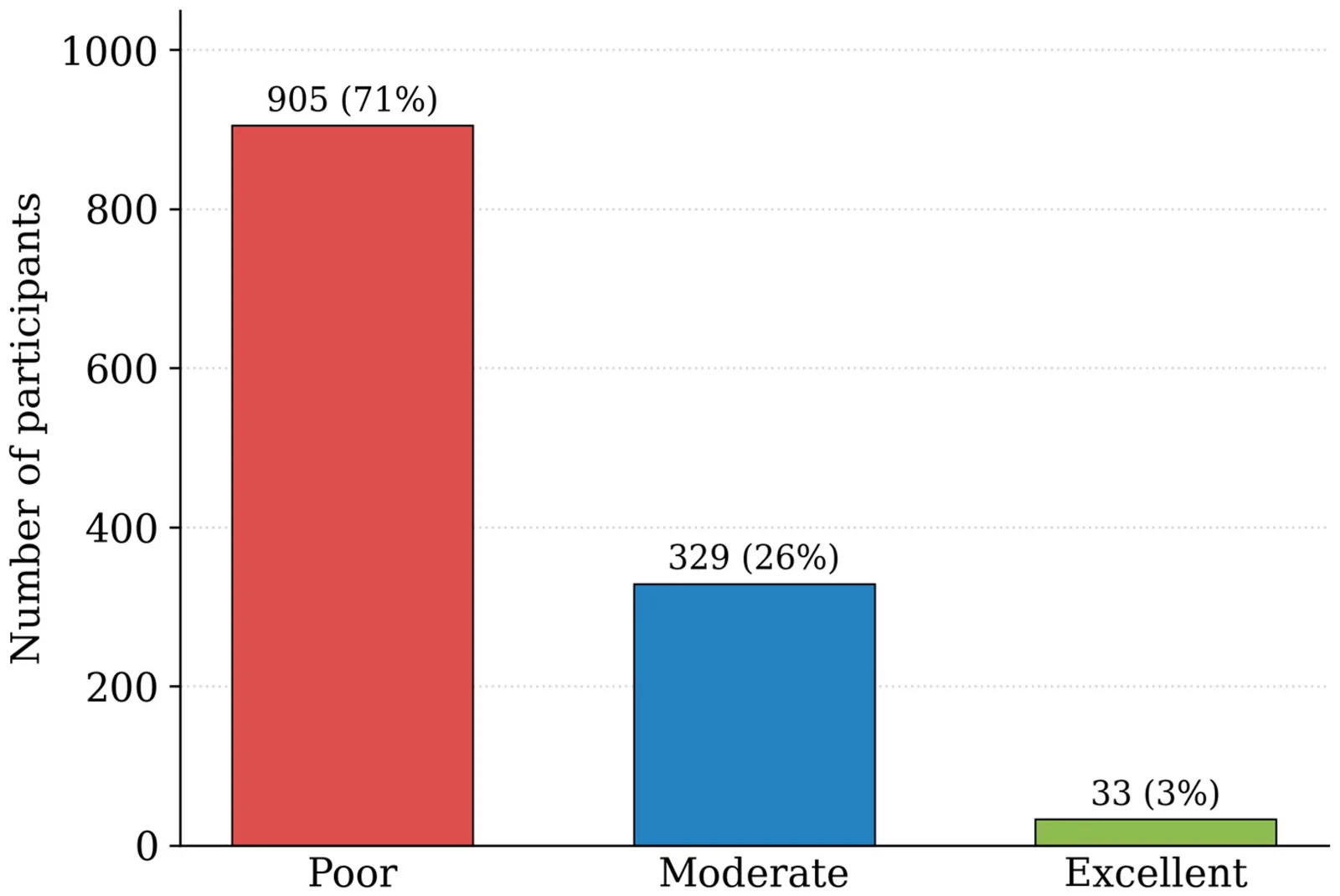
Knowledge Categories about Lumbar Puncture among the Sample (n = 1267).

**Figure 2:**
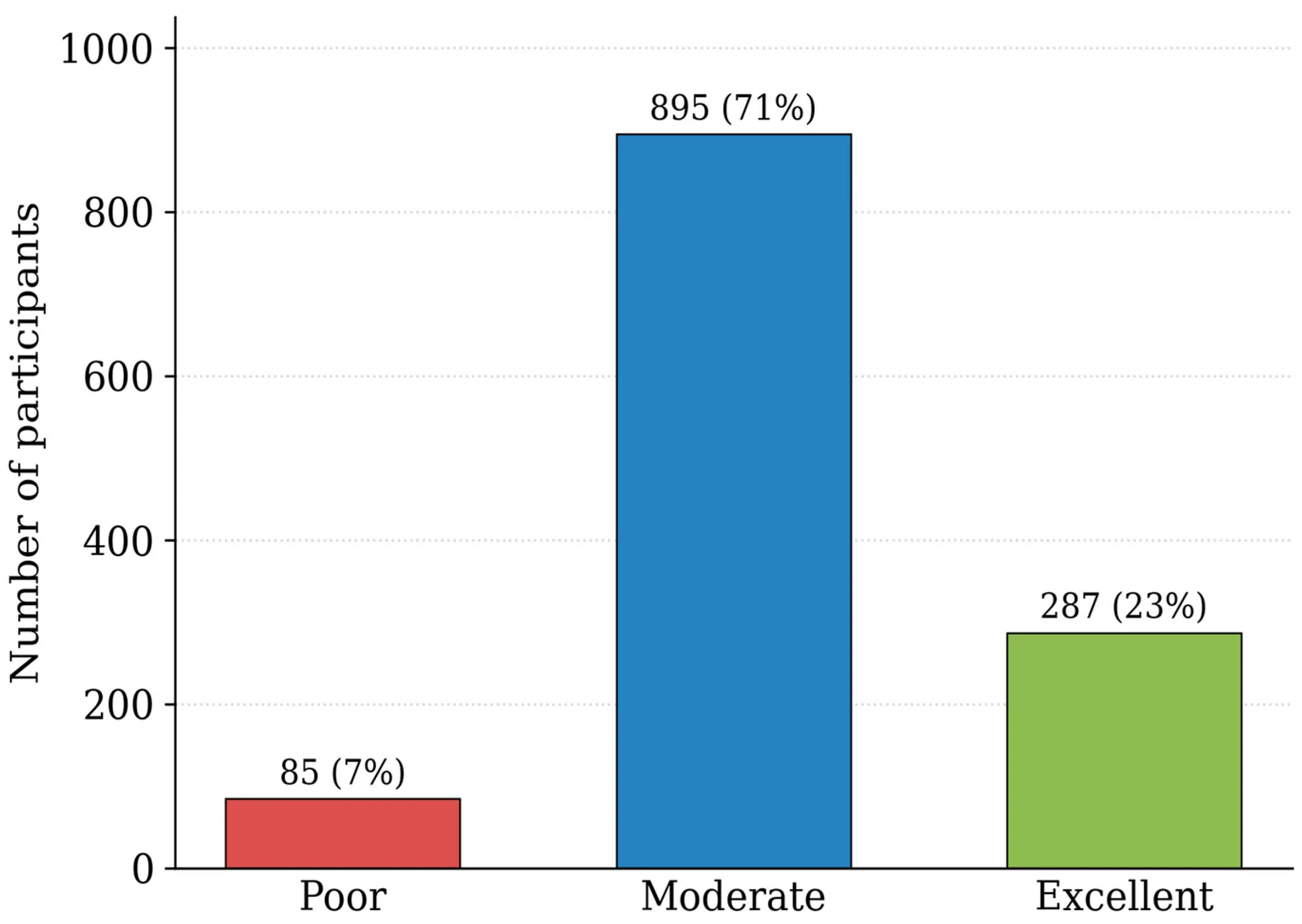
Attitude Categories about Lumbar Puncture among the Sample (n = 1267).

The mean knowledge score was 6.1 ± 3.9. Good knowledge (≥50%) was observed in 362 (29%) participants, of whom 33 (3%) demonstrated excellent knowledge and 329 (26%) had moderate knowledge; the remaining 905 (71%) participants had poor knowledge. The mean attitude score was 32.9 ± 5.5 out of a possible 10 to 50, corresponding to 66% of the maximum. Using the pre-specified thresholds, 287 (23%) were classified as excellent, 895 (71%) as moderate, and 85 (7%) as poor, giving 1182 (93%) at or above the 50% cut-off. This figure should be read against the arithmetic of the scale rather than as evidence of widespread enthusiasm, since 540 participants (43%) scored at or below 30 out of 50, the total produced by answering neutrally to every item, yet fell above the cut-off (Table 3, Figure 1 and Figure 2).

At the item level, while most participants correctly identified the LP image (953, 75%) and insertion site (784, 62%), conceptual understanding was inconsistent. Although 818 (65%) correctly defined LP, large proportions selected “don’t know” for key aspects such as analgesia (629, 50%), procedural duration (739, 58%), and diagnostic role (630, 50%). Misconceptions about complications were common: many participants were unsure about risks such as back pain (741, 58%), urinary incontinence (951, 75%), and erectile dysfunction (989, 78%). Notably, only 116 (9%) of participants correctly recognized that LP is not associated with urinary incontinence, underscoring the depth of this specific misconception. Overall, 801 (63%) were uncertain about LP safety (full item-level breakdown in Appendix A Table A2).

### 3.4. Attitudes Toward Lumbar Puncture

Table 4 shows attitude findings toward lumbar puncture among participants. A considerable proportion believed that society may not fully accept LP, with 649 (51%) agreeing/strongly agreeing, while 499 (39%) remained neutral. Nearly half were unsure about LP safety (618, 49%), though more participants disagreed (384, 30%) than agreed (265, 21%) that LP is unsafe. Awareness gaps were evident, as 727 (58%) agreed that society lacks understanding of LP’s importance. Encouragingly, most participants rejected the notion that education is unnecessary (662, 52%). Decision-making attitudes showed some hesitation, with 504 (40%) neutral about preferring discharge over LP. However, strong support existed for patient rights: 819 (65%) wanted an explanation, and 802 (63%) strongly supported informed consent. Willingness to proceed with LP was moderate, with 692 (55%) agreeing/strongly agreeing.

### 3.5. Relationship Between Knowledge and Attitude

Knowledge and attitude scores were positively correlated (Pearson r = 0.21, 95% CI 0.16 to 0.26, *p* < 0.001; Spearman rho = 0.20, *p* < 0.001) (Table 5A). Mean attitude scores rose across knowledge categories, from 32.4 ± 4.8 in the poor group to 33.7 ± 6.8 in the moderate group and 37.4 ± 4.9 in the excellent group (Table 5B). Cross-tabulation of the dichotomized categories was significant in the opposite direction, with 322 of 362 participants with good knowledge (89%) above the attitude cut-off compared with 860 of 905 with poor knowledge (95%) (χ^2^ = 14.3, *p* < 0.001) (Table 5C). In a sensitivity analysis restricted to the 11 knowledge items with unambiguous answers, the correlation was r = 0.22 (*p* < 0.001).

### 3.6. Determinants of LP Knowledge

Table 6 shows the determinants of higher knowledge of lumbar puncture. Male participants had lower knowledge scores (β = −0.50, 95% CI −0.96 to −0.03, *p* = 0.036). Higher educational attainment remained a strong predictor, with bachelor/diploma holders (β = 0.72, *p* = 0.004) and those with postgraduate education (β = 1.67, *p* = 0.001) demonstrating higher knowledge. Relative to unemployed respondents, those working in the health sector scored substantially higher (β = 3.25, *p* < 0.001), as did students (β = 2.08, *p* < 0.001) and those in the education sector (β = 1.26, *p* = 0.014). Higher income (>15,000 SAR) remained associated with increased knowledge (β = 0.95, *p* = 0.002). Other variables were not statistically significant. The model was significant overall (F (25, 1241) = 5.81, *p* < 0.001; R^2^ = 0.105, adjusted R^2^ = 0.087; AIC = 6919.3).

### 3.7. Determinants of Lumbar Puncture Attitude

Table 7 shows the determinants of lumbar puncture attitude, with the knowledge score entered as an explanatory variable. Knowledge was independently associated with attitude after adjustment for all sociodemographic and clinical covariates (β = 0.25 per additional correct item, 95% CI 0.17 to 0.33, *p* < 0.001), corresponding to approximately one additional attitude point for each standard deviation increase in knowledge. Post-graduate education (β = 1.85, *p* = 0.010) and income above 15,000 SAR (β = 1.27, *p* = 0.004) were also associated with more favorable attitudes, while higher BMI was associated with slightly less favorable attitudes (β = −0.08, *p* = 0.008). No occupational category reached significance. Adding the knowledge score improved model fit substantially (partial F (1, 1240) = 39.17, *p* < 0.001; AIC 7859.9 to 7822.5; R^2^ 0.065 to 0.093), and the full model was significant overall (F (26, 1240) = 4.91, *p* < 0.001; adjusted R^2^ = 0.074).

## 4. Discussion

### 4.1. Overview of Key Findings

This study assessed knowledge, attitudes, and misconceptions regarding lumbar puncture among adults in Saudi Arabia. Knowledge was limited, with 29% demonstrating good understanding of the procedure, its indications, and its complications, while attitude scores were comparatively favorable against thresholds that are lenient. Knowledge and attitude were positively related at the individual level. Needle-related fear was prevalent, and fear of insertion in the back was more common than fear of injection in the hand, which may reflect the perceived proximity of the spine to the central nervous system. Higher knowledge was observed among participants with higher educational attainment, students, and those working in healthcare or with higher household income, a pattern consistent with exposure-driven rather than innate awareness. This overall picture matches earlier work: a 2019 Riyadh study reported low knowledge alongside acceptable attitudes [8], a further Saudi study reported low knowledge with higher refusal than observed here [14], and a 2025 study among patients and caregivers in rural Zambia found that despite low median knowledge more than two-thirds would undergo the procedure if a doctor recommended it [15].

### 4.2. The Relationship Between Knowledge and Attitude

Aggregate figures invite a reading of low knowledge alongside favorable attitudes, but the individual-level analysis does not support treating the two as independent. Knowledge and attitude were significantly positively correlated (r = 0.21), knowledge remained an independent predictor of attitude after full adjustment, and mean attitude scores rose across knowledge categories. The dichotomized cross-tabulation ran counter to this pattern; at a threshold that 93% of the sample clears, the binary variable has little room to vary and is driven by the small minority at the lower end, so the continuous scores are the more informative comparison. The association is nevertheless weak, accounting for roughly 4% of the variance, so knowledge is best understood as one modest contributor to acceptance rather than its main determinant. This magnitude is closely comparable with earlier work. The Malaysian study that generated the source instrument reported a correlation of 0.272 (*p* = 0.001) [1], and the Riyadh study likewise found that participants with greater knowledge held more acceptable attitudes [8]; our estimate in a larger and more heterogeneous sample therefore replicates rather than contradicts these findings, and the consistency of a weak positive association across three populations argues that the relationship is real but limited in size. Alternative explanations for the favorable scores deserve equal weight. Most participants supported physician explanation and informed consent, consistent with previous studies reporting higher acceptability when consent is appropriately obtained [5,13,16], which suggests reliance on physician guidance despite limited personal understanding. Favorable scores are also prone to social desirability and acquiescence bias, particularly for items framed around deference to physician authority, and may reflect broader cultural deference documented in Saudi clinical settings [12] rather than specific confidence in the procedure.

### 4.3. Misconceptions About Indications and Complications

Substantial misconceptions were identified regarding the indications, safety, and complications of the procedure. Although 75% and 62% of participants correctly identified its appearance and needle insertion site, knowledge of clinical indications was lower: half were unaware of its role in diagnosing meningitis and encephalitis, 56% were unaware of its role in spinal anesthesia, and 47% were unaware of its therapeutic applications. The gap most likely to carry clinical consequence is the first, since unfamiliarity with the diagnostic role in suspected central nervous system infection is the scenario in which delayed consent could most directly translate into delayed diagnosis. Limited public exposure offers a plausible explanation, as the procedure is reserved for serious neurological illness and is far less familiar than blood tests or imaging.

Misconceptions about safety and complications were more pronounced. More than half were uncertain whether the procedure causes severe back pain, and most were uncertain about urinary incontinence (75%) or erectile dysfunction (78%); only 9% correctly recognized that urinary incontinence is not an expected complication. Sixty-three percent did not know whether the procedure is generally safe, and 66% were unaware that post-lumbar-puncture headache is the most common adverse event. Comparable misconceptions were reported in earlier work, including the belief that computed tomography or magnetic resonance imaging can replace the procedure entirely. These findings indicate a distorted understanding in which rare or non-existent complications are overestimated while common, self-limited ones go unrecognized.

### 4.4. Determinants of Knowledge and Attitude

Several sociodemographic factors were associated with knowledge, whereas attitudes were considerably less sensitive to participant characteristics. Higher educational attainment, student status, and employment in the health sector predicted better knowledge, underscoring the roles of formal education and occupational exposure; health-sector employment and higher income were similarly identified as predictors in earlier research [5,8,17], supporting the view that healthcare exposure and socioeconomic access to information are key modifiable drivers. Higher household income was also independently associated with better knowledge, which may reflect disparities in access to health information. Male sex was associated with slightly lower knowledge, a difference of half a point on an 18-point scale that is unlikely to be individually meaningful. Beyond knowledge itself, only postgraduate education and higher income were associated with attitude.

### 4.5. Implications for Practice and Research

Three implications follow. First, because knowledge explains only a small share of the variance in attitude, education alone should not be expected to change acceptance substantially, and interventions aimed purely at factual content such as pamphlets or awareness campaigns are unlikely to be sufficient on their own. Second, the pattern of misconception matters more than its volume: rare or non-existent complications were overestimated while common, self-limited ones went unrecognized, so correcting misplaced risk perception is a more useful target than raising general awareness. Third, because attitudes were largely insensitive to sociodemographic characteristics while support for physician explanation was near-universal, strengthening structured physician-patient communication at the point of consent is likely to influence real-world uptake more than population-level campaigns, with priority given to those with lower educational attainment and no healthcare exposure, in whom knowledge was lowest. Future work should test whether brief pre-procedural counseling improves both understanding and consent, and should employ probability sampling and a validated attitude instrument.

### 4.6. Strengths and Limitations

This study has several strengths. The sample was large, substantially exceeding the minimum calculated requirement, and drew on diverse sociodemographic backgrounds across Saudi Arabia, allowing variability in age, education, occupation, and income. Multivariable regression permitted adjustment for multiple potential confounders and identification of independent predictors rather than unadjusted associations.

Several limitations should also be considered. The cross-sectional design precludes causal inference. Recruitment used open social media channels with no sampling frame, no invitation denominator, and no regional quotas, so no response rate can be computed and the percentages describe self-selected online respondents rather than national estimates. Half the sample were students, two-thirds held a diploma or bachelor’s degree, and three-quarters lived in urban areas, all of which would be expected to bias knowledge estimates upward. The instrument also constrains interpretation. The anatomical-site item refers to insertion in the spinal cord, whereas insertion is made into the lumbar subarachnoid space below the termination of the cord; the item was presented as a choice between three anatomical images, but the wording requires revision before reuse. Items 10 to 16 are phrased without reference to probability and were scored dichotomously despite being context-dependent, although a sensitivity analysis restricted to the 11 unambiguous items reproduced every significant predictor and the knowledge-attitude association. The attitude scale combines societal perceptions, views on education, ethical expectations, personal willingness, and religious influence, which are not guaranteed to form a single construct; internal consistency was modest, so the composite should be read alongside the item-level distributions. Finally, the models explained a limited proportion of the variance, and unmeasured determinants such as prior clinical exposure, health literacy, and sources of health information warrant future study.

## 5. Conclusions

This study demonstrates that knowledge of lumbar puncture among self-selected Saudi adult respondents remains insufficient, with considerable misconceptions surrounding its indications, safety profile, and complications. Attitudes were comparatively favorable, reflecting support for informed consent and medical guidance, although the classification thresholds are lenient and a substantial minority responded neutrally throughout. Knowledge and attitude were weakly but significantly related, indicating a modifiable educational gap while suggesting that education alone is unlikely to be sufficient. Educational attainment, healthcare-sector employment, student status, and higher income were associated with better knowledge, underscoring the importance of health literacy and access to reliable information. Targeted educational initiatives and structured physician-patient communication are recommended to address misconceptions and support informed decision-making.

## Figures and Tables

**Table 1:** Sociodemographic characteristics of study participants (N = 1267).

Variable	Category	Frequency N (%)
Gender	Female	722 (57%)
	Male	545 (43%)
Nationality	Saudi	1156 (91%)
	Non-Saudi	111 (9%)
Age (Years)	Mean ± SD	30 ± 12
	Min–Max	18–73
	IQR (P25–P75)	20–39
Marital Status	Single	784 (62%)
	Married	443 (35%)
	Divorced/Widow	40 (3%)
Residence	Urban	960 (76%)
	Rural	307 (24%)
Education Level	Bachelor/Diploma	834 (66%)
	High school or lower	349 (28%)
	Postgraduate	84 (7%)
Employment	Student	631 (50%)
	Employed	404 (32%)
	Unemployed	232 (18%)
Monthly Household Income	<5000 SAR	405 (32%)
	5000–9999 SAR	296 (23%)
	10,000–14,999 SAR	224 (18%)
	≥15,000 SAR	342 (27%)

Abbreviations: N: Frequency; %: Percentages.

**Table 2:** Health-related Medical History of the Study Participants (n = 1267).

Condition	Response	Frequency N (%)
Diabetes Mellitus	No	1161 (92%)
	Yes	106 (8%)
Hypertension	No	1167 (92%)
	Yes	100 (8%)
Hyperlipidemia	No	1137 (90%)
	Yes	130 (10%)
Asthma	No	1173 (93%)
	Yes	94 (7%)
Sickle Cell Anemia	No	1240 (98%)
	Yes	27 (2%)
Thalassemia	No	1255 (99%)
	Yes	12 (1%)
Hyperthyroidism	No	1240 (98%)
	Yes	27 (2%)
Hypothyroidism	No	1209 (95%)
	Yes	58 (5%)
Rheumatoid Arthritis	No	1242 (98%)
	Yes	25 (2%)
Hernia	No	1228 (97%)
	Yes	39 (3%)

Abbreviations: N: Frequency; %: Percentages.

**Table 3:** Disc Knowledge and Attitude Scores and Categories about Lumbar Puncture (LP) (n = 1267).

Variable	Category	Frequency N (%)/Value
Knowledge Score	Mean ± SD	6.1 ± 3.9
Knowledge (Binary)	Good	362 (29%)
	Poor	905 (71%)
Knowledge (Category)	Excellent	33 (3%)
	Moderate	329 (26%)
	Poor	905 (71%)
Attitude (Binary)	Good	1182 (93%)
	Poor	85 (7%)
Attitude (Category)	Excellent	287 (23%)
	Moderate	895 (71%)
	Poor	85 (7%)

**Table 4:** Frequency Distribution of Responses Regarding Attitude toward Lumbar Puncture (LP) (n = 1267).

Item	Response	Frequency N (%)
Society still cannot accept LP completely	Strongly agree	288 (23%)
	Agree	361 (28%)
	Neutral	499 (39%)
	Disagree	85 (7%)
	Strongly disagree	34 (3%)
LP is not safe and should be avoided	Strongly agree	103 (8%)
	Agree	162 (13%)
	Neutral	618 (49%)
	Disagree	265 (21%)
	Strongly disagree	119 (9%)
Society doesn’t know the importance of LP	Strongly agree	324 (26%)
	Agree	403 (32%)
	Neutral	418 (33%)
	Disagree	85 (7%)
	Strongly disagree	37 (3%)
Society doesn’t need more education about LP	Strongly agree	107 (8%)
	Agree	105 (8%)
	Neutral	393 (31%)
	Disagree	371 (29%)
	Strongly disagree	291 (23%)
Would prefer discharge at own risk over LP	Strongly agree	115 (9%)
	Agree	140 (11%)
	Neutral	504 (40%)
	Disagree	293 (23%)
	Strongly disagree	215 (17%)
Wants doctor to explain the procedure	Strongly agree	594 (47%)
	Agree	225 (18%)
	Neutral	347 (27%)
	Disagree	63 (5%)
	Strongly disagree	38 (3%)
Informed consent should be obtained	Strongly agree	574 (45%)
	Agree	228 (18%)
	Neutral	375 (30%)
	Disagree	56 (4%)
	Strongly disagree	34 (3%)
Would sign consent if doctor recommends LP	Strongly agree	400 (32%)
	Agree	292 (23%)
	Neutral	480 (38%)
	Disagree	60 (5%)
	Strongly disagree	35 (3%)
Prefer other treatment over LP for diagnosis	Strongly agree	159 (13%)
	Agree	159 (13%)
	Neutral	529 (42%)
	Disagree	233 (18%)
	Strongly disagree	187 (15%)
Would refuse LP due to religious/elder belief	Strongly agree	105 (8%)
	Agree	115 (9%)
	Neutral	452 (36%)
	Disagree	271 (21%)
	Strongly disagree	324 (26%)

Abbreviations: N: Frequency; %: Percentages; LP: Lumbar puncture.

**Table 5:** Relationship between knowledge and attitude scores (n = 1267).

A. Correlation Between Continuous Scores	Coefficient (95% CI)	*p*
Pearson r, 18-item knowledge score	0.21 (0.16 to 0.26)	<0.001
Spearman rho, 18-item knowledge score	0.20	<0.001
Pearson r, 11-item sensitivity score	0.22 (0.17 to 0.27)	<0.001
**B. Attitude score by knowledge category**	**n (%)**	**Attitude score, mean ± SD**
Poor knowledge (<50%)	905 (71.4)	32.4 ± 4.8
Moderate knowledge (50–75%)	329 (26.0)	33.7 ± 6.8
Excellent knowledge (>75%)	33 (2.6)	37.4 ± 4.9
One-way ANOVA across categories	F(2, 1264) = 18.41	<0.001
**C. Cross-tabulation of dichotomized categories**	**Good attitude, n (%)**	**Poor attitude, n (%)**
Good knowledge (≥50%)	322 (89.0)	40 (11.0)
Poor knowledge (<50%)	860 (95.0)	45 (5.0)
Pearson chi-square	χ^2^ (1) = 14.30	<0.001

**Table 6:** Determinants of Lumbar Puncture Knowledge.

Predictor	Estimate (β)	95% CI	*p*
(Intercept)	4.00	1.98–6.03	<0.001
Age	0.02	−0.01–0.05	0.194
Sex [Male]	−0.50	−0.96–−0.03	0.036
Nationality [Saudi]	−0.30	−1.07–0.46	0.434
BMI	−0.00	−0.04–0.04	0.816
Marital Status [Married]	−0.39	−1.62–0.84	0.533
Marital Status [Single]	−0.14	−1.43–1.15	0.828
Residence [Urban]	−0.12	−0.62–0.37	0.622
Education Level [Bachelor/Diploma]	0.72	0.23–1.21	0.004
Education Level [Postgraduate]	1.67	0.69–2.65	<0.001
Role [Student]	2.08	1.18–2.99	<0.001
Role [Health sector]	3.25	2.24–4.26	<0.001
Role [Education sector]	1.26	0.25–2.26	0.014
Role [Administrative sector]	0.89	−0.39–2.16	0.172
Role [Military sector]	1.01	−0.31–2.34	0.132
Role [Industrial sector]	1.79	−0.48–4.05	0.123
Role [Handicrafts/Self-employment]	−0.60	−2.17–0.98	0.458
Role [Other]	0.64	−0.30–1.59	0.183
Monthly Income [5000–9999 SAR]	0.03	−0.54–0.60	0.916
Monthly Income [10,000–14,999 SAR]	−0.02	−0.66–0.61	0.939
Monthly Income [>15,000 SAR]	0.95	0.36–1.55	0.002
Smoking Status [Ex-smoker]	−0.27	−1.01–0.46	0.465
Smoking Status [Current smoker]	−0.39	−1.09–0.31	0.279
DM [Yes]	0.15	−0.73–1.02	0.744
HTN [Yes]	0.51	−0.42–1.44	0.282
Hyperlipidemia [Yes]	−0.34	−1.14–0.45	0.396

Observations = 1267; R^2^ = 0.105; adjusted R^2^ = 0.087. Abbreviations: CI: Confidence interval; BMI: Body mass index; SAR: Saudi Riyal; DM: Diabetes mellitus; HTN: Hypertension. *p* < 0.05 indicates statistical significance. Employment status and occupation were combined into a single role variable to remove the structural overlap between them. Reference categories: Sex = Female, Nationality = Non-Saudi, Marital Status = Divorced/Widow, Residence = Rural, Education = High school or lower, Role = Unemployed, Income ≤ 5000 SAR, Smoking = Never smoker.

**Table 7:** Determinants of Lumbar Puncture Attitude, Including Knowledge Score.

Predictor	Estimate (β)	95% CI	*p*
(Intercept)	32.51	29.61–35.42	<0.001
Knowledge score (per correct item)	0.25	0.17–0.33	<0.001
Age	−0.00	−0.04–0.04	0.967
Sex [Male]	−0.07	−0.74–0.59	0.833
Nationality [Saudi]	−0.17	−1.26–0.92	0.765
BMI	−0.08	−0.13–−0.02	0.008
Marital Status [Married]	−0.34	−2.09–1.42	0.706
Marital Status [Single]	0.75	−1.09–2.60	0.424
Residence [Urban]	−0.42	−1.13–0.29	0.242
Education Level [Bachelor/Diploma]	0.56	−0.14–1.26	0.114
Education Level [Postgraduate]	1.85	0.44–3.26	0.010
Role [Student]	0.13	−1.17–1.43	0.848
Role [Health sector]	0.28	−1.18–1.75	0.706
Role [Education sector]	−1.41	−2.85–0.03	0.055
Role [Administrative sector]	−0.25	−2.07–1.57	0.787
Role [Military sector]	−0.63	−2.52–1.26	0.512
Role [Industrial sector]	1.03	−2.21–4.27	0.532
Role [Handicrafts/Self-employment]	1.25	−0.99–3.50	0.274
Role [Other]	0.16	−1.20–1.51	0.819
Monthly Income [5000–9999 SAR]	0.64	−0.17–1.46	0.123
Monthly Income [10,000–14,999 SAR]	0.76	−0.14–1.66	0.099
Monthly Income [>15,000 SAR]	1.27	0.42–2.12	0.004
Smoking Status [Ex-smoker]	−0.46	−1.52–0.59	0.388
Smoking Status [Current smoker]	−0.83	−1.83–0.17	0.105
DM [Yes]	0.71	−0.54–1.95	0.268
HTN [Yes]	−0.44	−1.77–0.89	0.515
Hyperlipidemia [Yes]	0.11	−1.02–1.25	0.843

Observations = 1267; R^2^ = 0.093; adjusted R^2^ = 0.074. Abbreviations: CI: Confidence interval; BMI: Body mass index; SAR: Saudi Riyal; DM: Diabetes mellitus; HTN: Hypertension. *p* < 0.05 indicates statistical significance. Reference categories as in Table 6.

## Data Availability

The data presented in this study is available on request from the corresponding author. The data is not publicly available due to ethical restrictions and privacy concerns related to sensitive health information.

## Supplementary Materials

The following supporting information can be downloaded at: https://www.mdpi.com/article/10.3390/healthcare14162518/s1, STROBE Checklist.

## Abbreviations

The following abbreviations are used in this manuscript:

LP	Lumbar Puncture
CSF	Cerebrospinal fluid
R^2^	Coefficient of determination
CT	Computed tomography
MRI	Magnetic resonance imaging
OT	Operating theatre
OR	Odds Ratio
SAR	Saudi Arabian Riyal

## Appendix A

**Table A1:** Subjective Knowledge and Experience about Lumbar Puncture (LP) (n = 1267).

Item	Response	Frequency N (%)
Heard of LP	Yes	610 (48%)
	No	657 (52%)
Personal Experience with LP	Yes	33 (3%)
	No	1234 (97%)
Family/Friends Experience	Yes	170 (13%)
	No	1097 (87%)
Fear of Needle (Hand Injection)	Yes	375 (30%)
	No	892 (70%)
Fear of Needle (Back Injection)	Yes	877 (69%)
	No	390 (31%)

Abbreviations: N: Frequency; %: Percentages.

**Table A2:** Frequency Distribution of Responses Regarding Knowledge of Lumbar Puncture (LP) (n = 1267).

	Item *	Response	Frequency N (%)
1	Which photo shows the LP procedure?	A	110 (9%)
		B	953 (75%)
		C	203 (16%)
2	Which part of the spinal cord is the needle inserted?	A	108 (9%)
		B	374 (30%)
		C	784 (62%)
3	Which photo showed the right LP procedure?	A	836 (66%)
		B	430 (34%)
4	LP is a procedure to withdraw brain fluid via the lower back	Yes	818 (65%)
		No	40 (3%)
		Don’t know	409 (32%)
5	Analgesics/sedatives make LP relatively painless	Yes	474 (37%)
		No	164 (13%)
		Don’t know	629 (50%)
6	Procedure takes ~10 min in the ward, no OT needed	Yes	427 (34%)
		No	101 (8%)
		Don’t know	739 (58%)
7	LP diagnoses bacterial/viral/fungal CNS infections	Yes	586 (46%)
		No	51 (4%)
		Don’t know	630 (50%)
8	LP is done to give spinal anaesthetic	Yes	450 (36%)
		No	108 (9%)
		Don’t know	709 (56%)
9	LP is used therapeutically (e.g., chemotherapy)	Yes	586 (46%)
		No	81 (6%)
		Don’t know	600 (47%)
10	LP can cause severe back pain	Yes	407 (32%)
		No	119 (9%)
		Don’t know	741 (58%)
11	After LP, patient may have urinary incontinence	Yes	200 (16%)
		No	116 (9%)
		Don’t know	951 (75%)
12	After LP, patient may have erectile dysfunction	Yes	148 (12%)
		No	130 (10%)
		Don’t know	989 (78%)
13	CT scan done before LP if contraindication suspected	Yes	395 (31%)
		No	72 (6%)
		Don’t know	800 (63%)
14	CT/MRI can replace LP for accurate diagnosis	Yes	259 (20%)
		No	162 (13%)
		Don’t know	846 (67%)
15	LP causes severe complications	Yes	418 (33%)
		No	118 (9%)
		Don’t know	731 (58%)
16	LP is a low-risk, relatively safe procedure	Yes	283 (22%)
		No	183 (14%)
		Don’t know	801 (63%)
17	Commonest LP complication is post-puncture headache	Yes	371 (29%)
		No	62 (5%)
		Don’t know	834 (66%)
18	Headache prevented by lying flat 6h after LP	Yes	283 (22%)
		No	63 (5%)
		Don’t know	921 (73%)

Abbreviations: N: Frequency; %: Percentages; LP: Lumbar puncture; CT: Computed tomography; MRI: Magnetic resonance imaging. * Items 1 to 3 were presented to participants as labelled illustrations. Item 1: A, pericardiocentesis; B, lumbar puncture (correct); C, thoracentesis. Item 2: a sagittal diagram of the brain and spinal cord with pointers at the cervical (A), thoracic (B) and lumbar (C) levels, with C correct. Item 3: A, needle inserted into the lumbar subarachnoid space (correct); B, bone marrow biopsy.
