# Supplementary material for: Lumbar Puncture in Saudi Arabia: Evaluating Knowledge, Attitude and Sociodemographic Influences
Source: Healthcare (Basel). 2026 Aug 12;14(16):2518. doi: 10.3390/healthcare14162518 (PMC13511820; doi:10.3390/healthcare14162518)
Supplement: Supplementary file 1 [file healthcare-14-02518-s001.zip › healthcare-4461853-supplementary.pdf]

STROBE Checklist

Checklist of items that should be included in reports of cross-sectional studies.

Section / Topic	Item No	Recommendation	Page No
**Title and abstract**	1(a)	Indicate the study's design with a commonly used term in the title or the abstract	1
	1(b)	Provide in the abstract an informative and balanced summary of what was done and what was found	1–2
**Introduction**			
Background/rationale	2	Explain the scientific background and rationale for the investigation being reported	2–3
Objectives	3	State specific objectives, including any prespecified hypotheses	3
**Methods**			
Study design	4	Present key elements of study design early in the paper	4
Setting	5	Describe the setting, locations, and relevant dates, including periods of recruitment, exposure, follow-up, and data collection	4, 5
Participants	6	Cross-sectional study; Give the eligibility criteria, and the sources and methods of selection of participants	4
Variables	7	Clearly define all outcomes, exposures, predictors, potential confounders, and effect modifiers. Give diagnostic criteria, if applicable	5
Data sources / measurement	8	For each variable of interest, give sources of data and details of methods of assessment (measurement)	5
Bias	9	Describe any efforts to address potential sources of bias	4, 5, 22
Study size	10	Explain how the study size was arrived at	4
Quantitative variables	11	Explain how quantitative variables were handled in the analyses; if applicable, describe which groupings were chosen and why	5
Statistical methods	12(a)	Describe all statistical methods, including those used to control for confounding	6
	12(b)	Describe any methods used to examine subgroups and interactions [No subgroup or interaction analyses were performed.]	Not applicable
	12(c)	Explain how missing data were addressed	6
	12(d)	Describe analytical methods taking account of sampling strategy (cross-sectional)	4, 6
	12(e)	Describe any sensitivity analyses	6, 16
**Results**			
Participants	13(a)	Report numbers of individuals at each stage of study, eg numbers potentially eligible, examined for eligibility, confirmed eligible, included in the study,	4, 7, 22

		and analysed [1,267 responses were collected and all were analyzed. No sampling frame or eligibility screening denominator was available; see page 21.]	
	13(b)	Give reasons for non-participation at each stage [The questionnaire link was distributed through open social media channels, so no invitation denominator or record of non-participation exists. Stated as a limitation on page 21.]	Not applicable
	13(c)	Consider use of a flow diagram [A flow diagram is not informative without an invitation denominator; the analytic sample of 1,267 is reported on page 7.]	Not applicable
Descriptive data	14(a)	Give characteristics of study participants (eg demographic, clinical, social) and information on exposures and potential confounders	7–9; Tables 1–2
	14(b)	Indicate number of participants with missing data for each variable of interest [Three image-based knowledge items were unanswered by one participant; all other variables were complete for all 1,267 respondents.]	6
Outcome data	15	Cross-sectional study; Report numbers of outcome events or summary measures	10–16, 25–26; Tables 3–5, Figures 1–2, Appendix Tables S1–S2
Main results	16(a)	Give unadjusted estimates and, if applicable, confounder-adjusted estimates and their precision (95% CI). Make clear which confounders were adjusted for and why they were included [Confounder-adjusted estimates with 95% CIs are reported in Tables 5 and 6; unadjusted estimates were not tabulated.]	17–19; Tables 6–7
	16(b)	Report category boundaries when continuous variables were categorized	5, 10
	16(c)	If relevant, consider translating estimates of relative risk into absolute risk for a meaningful time period [Outcomes were continuous scores analyzed by linear regression, so no relative risk was estimated.]	Not applicable
Other analyses	17	Report other analyses done, e.g. analyses of subgroups and interactions, and sensitivity analyses	15–16
**Discussion**			
Key results	18	Summarise key results with reference to study objectives	20
Limitations	19	Discuss limitations of the study, considering sources of potential bias or imprecision. Discuss both direction and magnitude of any potential bias	22
Interpretation	20	Give a cautious overall interpretation of results considering objectives, limitations, multiplicity of analyses, results from similar studies, and other relevant evidence	20–22

Generalisability	21	Discuss the generalisability (external validity) of the study results	22
Other information			
Funding	22	Give the source of funding and the role of the funders for the present study	24
